# Magnitude and associated factors of intimate partner violence among youth women in Ethiopia: multilevel analysis based on 2016 Ethiopian Demographic and Health Survey

**DOI:** 10.1186/s12905-022-02143-9

**Published:** 2022-12-26

**Authors:** Nuhamin Tesfa Tsega, Daniel Gashaneh Belay, Fantu Mamo Aragaw, Melaku Hunie Asratie, Moges Gashaw, Mastewal Endalew

**Affiliations:** 1 Department of Women’s and Family Health, School of Midwifery, College of Medicine and Health Sciences, University of Gondar, Gondar, Ethiopia; 2 Department of Human Anatomy, College of Medicine and Health Sciences, University of Gondar, Gondar, Ethiopia; 3 Department of Epidemiology and Biostatistics, Institute of Public Health, College of Medicine and Health Sciences, University of Gondar, Gondar, Ethiopia; 4 Department of Physiotherapy, School of Medicine, College of Medicine and Health Science, University of Gondar, Gondar, Ethiopia; 5 Department of Environmental and Occupational Health and Safety, Institute of Public Health, College of Medicine and Health Sciences, University of Gondar, Gondar, Ethiopia

**Keywords:** Magnitude, Intimate partner violence, Multilevel analysis, Youth, Ethiopia

## Abstract

**Background:**

The period of youth is important for the foundation of healthy and stable relationships, women’s health and well-being. Youth women face a higher risk of experiencing violence than older women. Intimate partner violence (IPV) against youth women is a significant public health concern. Despite paramount negative health consequences of IPV for the survivor, as per our knowledge, research study on IPV and associated factors among youth women in Ethiopia is scarce. Therefore, this study aimed to assess the magnitude and associated factors of IPV among youth women in Ethiopia.

**Methods:**

The data was accessed from 2016 Ethiopia demographic and health survey (EDHS) which was a cross sectional population based household survey. It was also conducted using a multi-stage stratified random cluster sampling approach. The data were cleaned, weighted, and analyzed using STATA Version 14 software. The total weighted sample of 1077 youth women were used in this study. Multilevel logistic regression modeling was used to determine factors associated with IPV among youth women. Adjusted odds ratio (AOR) with 95% confidence interval (CI) and p value < 0.05 were used to declare the significant variables.

**Results:**

Among the total participants, 30.27% (95% CI 27.59, 33.09) of youth women experienced IPV. Individual level variables such as: Being widowed/divorced/separated (AOR = 2.28; 95% CI 1.33, 3.91), having a partner who drinks alcohol (AOR = 5.76; 95% CI 3.42, 9.69), witnessing inter-parental violence during childhood (AOR = 3.45; 95% CI 2.21, 5.37), being afraid of partners (AOR = 7.09; 95% CI 4.30, 11.68), and from community level variables, youth women residing in communities with a low proportion of educated youth women (AOR = 0.31; 95% CI 0.13, 0.78) were significantly associated with having experience of IPV.

**Conclusion:**

The magnitude of intimate partner violence among youth women in Ethiopia was relatively high as compared to the global estimate of IPV. Individual and community level variables such as currently widowed/divorced/separated women, having a partner who drinks alcohol, witnessing inter-parental violence, being afraid of partner, and women from a low proportion of community level youth women's education were significantly associated with intimate partner violence. To decrease this public health problem, it is better to strengthen legislation on the purchase and sale of alcohol, provide legal protection for separated/divorced women, establish effective legal response services for IPV, promote gender equality, and provide psychological support for those who witnessed inter-parental violence during childhood to reduce IPV.

## Background

Intimate partner violence (IPV) against women is a significant public health issue as well as a violation of human rights, with both short and long term consequences for women's physical, mental, sexual, and reproductive health [1]. The World Health Organization (WHO) defines IPV as a range of physical, sexual, or emotional/psychological abuse by a current or former partner [2].

Intimate partner violence has been recognized as a major medical, psychological, and social problem for the survivors. Women subjected to IPV tend to have other behavioral risk factors for chronic disease, and the impacts extend to the whole family, particularly in children who witnessed IPV [3]. It increases negative health consequences for maternal and child health such as; increase the risk of experiencing depression [4, 5], injury, post-traumatic stress disorder [6], adverse pregnancy outcomes [7], low birth weight and unintended pregnancy [8, 9]. It is a leading cause of suicidal behaviors [10] and increases the risk of HIV infection [11, 12]. Besides, an evidence showed that IPV contributes to more than 10.3% of violent deaths [13] and leads to substantial economic costs for governments, communities, and individuals [14].

Youth women face a higher risk of experiencing violence as compared to older women. The period of youth is important for the foundation of healthy and stable relationships, women’s health and well-being [15]. This population group is mostly affected by social and economic inequalities, making them potentially vulnerable to violence including IPV. Experiencing IPV at an early age can also increase the risk of adulthood exposure to IPV [16].

Globally, WHO estimated that 24% and 26% of women aged 15–19 and 20–24 years experienced lifetime IPV, respectively [17]. In India, the prevalence of IPV among youth women was 29% [18], and in Mozambique 60% of youth women had experienced IPV in their lifetime [19]. A study done in South Africa revealed that 13.1% of youth women experienced IPV [20]. In Sub-Saharan Africa (SSA), the prevalence of IPV among youth women ranges from 28.77% to 67% [21]. As per our knowledge, evidence regarding IPV among youth women in Ethiopia is scarce.

Evidences have shown that socio-demographic and other factors such as; Residency [20], educational status [22, 23], marital status [24, 25], occupational status [19], partner alcohol use [18, 26, 27], poverty [18], witnessed inter-parental violence [26, 28], wealth status [18, 20], afraid of partner [29–31] and acceptance of IPV [18] are the main factors associated with IPV among youth women. Evidence suggests that gender inequalities increase the vulnerability of violence men against women [32, 33].

Tackling IPV has a huge benefit in saving individual and societal costs as well as reducing the morbidity of youth women [34]. To achieve the Sustainable Development Goals (SDGs) by 2030, the international community committed to the achievement of gender equality and elimination of all forms of violence against women and girls. Promoting gender equality, preventing violence against women and girls (SDG goal 5), and ensuring responsive and inclusive societies (SDG target 16.1) are far-reaching SDG goals to ensure gender equity [35].

Despite the negative health consequences of IPV for the survivors of youth women, there is no study regarding the magnitude as well as individual and community level factors associated with IPV among youth women in Ethiopia at a national level. Therefore, this study aimed to assess the magnitude and individual and community level associated factors of IPV among youth women in Ethiopia. The finding from this study will give an insight for policymakers in understanding the burden of IPV among youth women and its associated factors for setting possible interventions and ensure or deliver safe and reliable service.

## Methodology

### Study design and setting

In this study, we used a population based cross-sectional survey data from the 2016 EDHS. Ethiopia is a sub-Saharan African country with 1.1 million square kilometers of coverage and the second most populous country in Africa with an estimated population of 100,613,986 people. Ethiopia's administrative structure is federally decentralized, with two city administrations and nine regions [36].

### Data source, study population, and sampling procedure

The current study was based on secondary data analysis of the 2016 EDHS, which was a national survey conducted between January 18, 2016, and June 27, 2016. The survey was carried out by the statistical agency in collaboration with the Ethiopian public health institute, the federal ministry of health, and inner city fund international, which provides technical assistance through its measure demographic and health survey project, a USAID-funded program that supports the implementation of population and health surveys in countries around the world. The survey used stratified cluster sampling, selected in two stages. A total of 645 clusters were chosen in the first stage, with 28 households chosen for each cluster in the second stage. For this study, we used the IR data set and the study population was youth women (aged 15–24 years) who had ever been married and completed the IPV questionnaire. A total weighted sample of 1077 youth women were included in our study.

### Variables of the study

The dependent variable of this study was IPV, which was a binary outcome variable coded as “0” if it was not violated and “1” if it was violated. Whereas the independent variables of this study were further classified into individual and community level variables. The individual-level variables used in this study were; youth age, youth education, youth occupation, marital status, husband/partners education, husband/partners occupation, household wealth status, media exposure, women afraid of husband/partner, women’s attitude towards wife beating, inter-parental violence witnessed, and women’s decision making power. Six community-level variables; residence, region, community level women's education, community level poverty, community level of youth women wife beating acceptance, and community-level media exposure were also used as independent variables in this study. The community-level variables such as community level women's education, community level poverty, community level of youth women wife beating acceptance and community-level media exposure were created by aggregating individual-level variables since these variables are not directly found from the survey.

### Operational definitions

#### Intimate partner violence

If the respondent said “Yes” to any one of the ranges of physical, sexual, and emotional violence or any combination of the three forms of violence committed by their current or former intimate partner/husband, it was considered as experienced IPV [37].

Physical violence was measured by ever-married youth women who have been experienced one or more of the following acts committed by their current or former intimate partner/husband; pushing, shaking, or throwing something at; slapping; punching with fist or hitting with something harmful; kicking, or dragging; trying to choke or burn on purpose; or threatening or attacking with knife, gun, or any other weapon and twisting arm or pulling hair. Sexual violence was also measured by asking ever-married youth women and answered “yes” to one or more of the following acts (forced into unwanted sex, forced into unwanted sexual acts, and physically forced to perform sexual acts). Similarly, to measure emotional violence in the survey, ever-married youth women experienced at least one of the following acts committed by their current or former intimate partner/husband (humiliation, harm, and insult or made them feel bad).

#### Acceptance of wife beating

Youth women were asked whether situations of beating a wife were justifiable in the following situations (if she goes without telling him, if she argues with him, if she burns the food, if she neglects the children, and if she refuses to have sex with him). If a woman answered "Yes" to one or more of the questions, it was considered she accepted a wife beating [38].

#### Youth women’s decision-making autonomy

The decision-making power is composed of four questions. The women were asked who usually decides on the respondent’s health care, large household purchases, what to do with the money their husband earns, and visits to her family or relatives. For each item, the response was given a score of 0–2. The total score was 8. Hence, those women who scored four and above were categorized as having high decision-making power [39].

#### Media exposure

Young women were considered to be exposed if they had been exposed to at least one of the three media (television, radio, or newspaper) and otherwise unexposed. Community level media exposure was measured by the proportion of youth women who have been exposed to at least one media (television, radio, or newspaper) in a cluster, and categorized and coded as “0” for low (communities in which < 13.8% of women had at least one media exposure) and “1” for high (communities in which ≥ 13.8% of women had at least one media exposure) based on the national median value [40]. Community level poverty was also determined by the proportion of youth women in the poorer and poorest quantiles obtained from the household wealth index result. Then, it was categorized and coded as “0” for low (communities in which < 24% of youth women had poorer and poorest wealth quantiles) and “1” for high ( communities in which ≥ 24% of youth women had poorer and poorest wealth quantiles) based on the national median value [41]. Community level youth women’s education was measured by the proportion of youth women who had at least primary level of education in a cluster. Then, it was classified and coded as “0” for low (communities in which < 7.7% of women who had at least primary education) and “1” for high (communities in which ≥ 7.7% of women who had at least primary education) using the national median value of community education [40]. Community level of youth women’s wife beating acceptance was determined by the proportion of youth women who had a wife-beating accepting attitude in a cluster and classified and coded as “0” for low (communities in which < 50% of women had a wife-beating accepting attitude) and “1” for high (communities in which ≥ 50% of women had a wife-beating accepting attitude) based on the national median value of wife beating acceptance since it was not normally distributed [42].

### Data processing and statistical analysis

Data extraction, coding, and analysis were done using Stata version 14. Throughout the study, data weighting was done by using the available sample weight factor to adjust for non-proportional sample selection and for non-responses as well as to restore the representativeness of the data. Multilevel logistic analysis was done because of the hierarchical nature of EDHS data and bivariable multilevel logistic regression analysis was performed to estimate the crude odds ratios at 95% CI and those variables with p-value ≤ 0.2 were considered for multivariable analysis. In the multivariable multilevel logistic regression analysis, those variables with p-vale < 0.05 were declared significantly associated with IPV. Multi-collinearity was also checked using the variance inflation factor (VIF) and indicates that there was no multi-collinearity since all variables have VIF < 5 [43].

We fitted four models that contained variables of interest. These models were model 1 (a null model), which was fitted without explanatory variables, model 2 (which only examined the effects of individual-level variables), model 3 (containing community-level variables only), and model 4 (containing both individual and community-level variables simultaneously). The Intra-class Correlation Coefficient (ICC), proportional change in variance (PCV), and median odds ratio (MOR) were used to examine the random effect. The calculation for MOR and PCV is as follows: ${\text{MOR}} = e^{{0.95}{\sqrt {VA} }}$ [44] where; VA is the area level variance; $PCV = \frac{Vnull - VA}{{V null}}*100\%$ [44] where; Vnull = variance of the initial model, and VA = variance of the final model. Model comparison/fitness was assessed by deviance, and the model with the lowest deviance was used as the best-fitted model.

### Ethical consideration

In this study, ethical approval was not required because a secondary data analysis was conducted based on the 2016 EDHS data, which was done with the government's permission, informed consent was obtained, and participant confidentiality was ensured at the time. Besides, for the sake of anonymity, the data set had no individual names or household addresses. For conducting the current study, the data sets were downloaded with permission from the “Measure DHS program” by requesting them after explaining the purpose of the study..

## Result

### Background characteristics of study participants

A total of 1077 ever married youth women were included in this analysis. Almost three-fourths of study participants were in the age group of 20–24 years old, with an overall mean age of 20.73 (SD ± 0.069) years old. Of the study participants, more than half (53.42%) of the respondents attained primary education. The majority (74.12%) of youth women witnessed inter-parental violence. Regarding the wealth index, 505 (46.94%) youth women had poor wealth status. Two-thirds of youth women had media exposure while the majority (84.93%) and (89.76%) of the study participants were from rural areas and large central regions, respectively (Table 1).

**Table 1:** Background characteristics of study participants in a study of magnitude and associated factors of IPV among youth women in Ethiopia based on 2016 EDHS

Variables	Category	Weighted frequency	Percentage (100%)
Age	15–19	290	26.96
	20–24	787	73.04
Religion	Orthodox	438	40.67
	Muslim	398	36.91
	Protestant	203	18.86
	Others	38	3.56
Currently marital status	Married	927	86.11
	Unmarried	150	13.89
Youth education status	No formal education	352	32.71
	Primary education	575	53.42
	Secondary and above	150	13.87
Husband/partner’s education	No formal education	308	32.23
	Primary education	459	48.05
	Secondary and above	188	19.72
Youth’s occupation	Unemployed	589	54.65
	Employee	488	45.35
Husband/partner occupation	Unemployed	71	7.46
	Employee	884	92.54
Wealth index	Poor	505	46.94
	Middle	189	17.53
	Riche	383	35.53
Partner/husband drinking alcohol	No	755	70.09
	Yes	322	29.91
Respondent afraid of partner	No	444	41.18
	Yes	633	58.82
Youth women’s decision making autonomy	Lower	286	30.12
	Higher	662	69.88
Youth women acceptance of WB	Unacceptance	349	32.43
	Acceptance	728	67.57
Witnessed inter-parental violence	No	798	74.12
	Yes	279	25.88
Media exposure	Unexposed	707	65.66
	Exposed	370	34.34
*Community level variables*			
Residency	Urban	162	15.03
	Rural	915	84.93
Region	Metropolis	38	3.53
	Large centrals	967	89.76
	Small peripherals	72	6.71
Community level media exposure	Low	417	38.71
	High	660	61.29
Community level of poverty	Low	374	34.68
	High	703	65.32
Community level of youth women’s education	Low	116	10.77
	High	961	89.23
Community level of youth women WB acceptance	Low	440	40.81
	High	637	59.19

*NB: WB* wife beating

### Magnitude of intimate partner violence among youth women in Ethiopia

The overall magnitude of IPV among youth women was 30.27% (95% CI 27.59, 33.09). The most prevalent form of IPV was physical violence (22.52%) and the least prevalent form of IPV was sexual violence (8.44%) (Table 2).

**Table 2:** Magnitude of intimate partner violence against youth women in Ethiopia: based on 2016 EDHS

Form of intimate partner violence	Weighted frequency	Magnitude (95% CI)
Physical violence	242	22.52% (20.12, 25.11)
Sexual violence	91	8.44% (6.91, 10.26)
Emotional violence	214	19.89 (17.61, 22.39)
Physical or sexual or emotional violence	326	30.27% (27.59, 33.09)

### Random effect and model comparison results

As indicated in details from Table 3, the value of ICC in the null model was 0.41, which means that about 41% of the variations in IPV among youth women were attributed to the difference at the cluster level, but the rest 59% were attributed to individual factors. The highest MOR (4.18) was revealed in the null model, which also indicated that the median odds of experiencing IPV between the lowest and the highest IPV clusters. Moreover, the PCV value in the final model (model 4) was 20%, which indicates the variation in the experience of IPV among youth womn was explained by both the individual and community-level variables simultaneously. The model comparison/fitness was done using deviance test, then the final model has the lowest deviance (1038) and was taken as the best fitted model (Table 3).

**Table 3:** Multilevel logistic regression analysis of individual and community level factors associated with IPV among youth women in Ethiopia: based on 2016 EDHS

Variables	Model 1	Model 2	Model 3	Model 4
		AOR (95% CI)	AOR (95% CI)	AOR (95% CI)
Current marital status				
Married	–	1	–	1
Widowed/divorced/separated	–	2.31 (1.35, 3.97)	–	2.28 (1.33, 3.91)**
Youth’s occupation				
Unemployed	–	0.85 (0.57, 1.27)	–	0.88 (0.59, 1.31)
Employee	–	1	–	1
Partner/husband drinking alcohol				
No	–	1	–	1
Yes	–	5.79 (3.43, 9.79)	–	5.76 (3.42, 9.69)***
Wealth index				
Poor	–	0.98 (0.55, 1.76)	–	1.14 (0.60, 2.14)
Middle	–	1.09 (0.58, 2.08)	–	1.17 (0.59, 2.31)
Riche	–	1	–	1
Witness inter-parental violence				
No	–	1	–	1
Yes	–	3.40 (2.19, 5.29)	–	3.45 (2.21, 5.37)***
Media exposure				
No	–	1	–	1
Yes	–	0.61 (0.38, 0.99)	–	0.66 (0.39, 1.12)
Respondent afraid of partner				
No	–	1	–	1
Yes	–	7.00 (4.26, 11.52)	–	7.09 (4.30, 11.68)***
Youth women acceptance of WB				
Unaccepted	–	1	–	1
Accepted	–	0.87 (0.55, 1.37)	–	0.86 (0.54, 1.36)
Community level variables				
Residency				
Urban	–	–	1	1
Rural	–	–	1.41 (0.72, 0.78)	0.84 (0.36, 1.93)
Community level media exposure				
Low	–	–	1	1
High	–	–	0.64 (0.37, 1.12)	0.71 (0.37, 1.39)
Community level of youth women’s education				
Low	–	–	0.33 (0.14, 0.75)	0.31 (0.13, 0.78)*
High	–	–	1	1
Random effect				
VA	2.26	2.21	2.16	1.89
ICC	0.41	0.40	0.39	0.36
MOR	4.18	4.11	4.04	3.69
PCV	Reff	0.02	0.04	0.20
Model comparison				
Deviance	1214	1046	1204	1038
Mean VIF	–	1.24	1.09	1.37

*ICC* inter cluster corrolation cofficent, *MOR* median odds ratio, *PCV* proportional change in variance, *AOR* adjusted odds ratio, *CI* confidence intervalm, *WB* wife beating

*P value < 0.05; **P  value < 0.01; ***Pvalue < 0.001

### Factors associated with intimate partner violence

In the bivariable multilevel logistic regression analysis: current marital status, youth’s occupation, partner/husband drinking alcohol, wealth index, witnessing inter-parental violence during childhood, respondents afraid of their partner, media exposure, and youth women’s acceptance of wife beating, residency, community-level media exposure, and community-level of youth women’s education were considered for the multivariable multilevel logistic analysis (P value ≤ 0.2). In the final model result, current marital status, partner/husband drinking alcohol, witnessing inter-parental violence during childhood, respondents afraid of their partner, and community level of youth women’s education were found to be significantly associated with IPV among youth women.

The odds of experiencing IPV were 2.28 (AOR = 2.28; 95% CI 1.33, 3.91) times higher among youth women who are widowed/divorced/separated youth women as compared to married youth women. Youth women who had a partner who drank alcohol were 5.76 times (AOR = 5.76; 95% CI 3.42, 9.69) more likely to report IPV compared to youth women who had a partner who did not drink alcohol. Regarding witnessing inter-parental violence during childhood, the odds of experiencing IPV among youth women who had witnessed inter-parental violence were 3.45 (AOR = 3.45; 95% CI 2.21, 5.37) times higher as compared with youth women who had not witnessed inter-parental violence. Furthermore, youth women who were afraid of their partner were 7.09 times (AOR = 7.09; 95% CI 4.30, 11.68) more likely to report IPV than their counterparts. The odds of experiencing IPV were reduced by 69% (AOR = 0.31; 95% CI 0.13, 0.78) among women from communities with a low proportion of educated youth women compared to women from communities with a high proportion of educated youth women (Table 3).

## Discussion

The current study was a population-based survey, which provides new information about IPV among youth women in Ethiopia and factors placing them at risk. According to this secondary data analysis, the prevalence of lifetime IPV experience among young women in Ethiopia was 30.27% (95% CI 27.59, 33.09), which is in line with a study conducted in India (29%) [18]. On the other hand, the finding of this study is lower than previous studies conducted in Mozambique (60%) [19], and Malawi (75%) [5]. The possible justification for the variation might be due to differences in study settings and methods of data collection. In our study, we used face-to-face interviews as the method of data collection, which might lead to under reporting of IPV because of social desirability bias. In contrast, a study conducted in Mozambique used self-administered questionnaires, which might provide more confidence for the participants to disclose their experiences of IPV.

However, the result of this study is higher than the prevalence reported in South Africa (13.1%) [20], Nigeria (21%) [28], and SSA 25.2% [45]. The possible discrepancy might be due to the difference in outcome variable measurement and study period. In our study, we assessed the lifetime prevalence of IPV and included either of physical, sexual, or emotional violence, whereas in SSA and Nigeria, they examine IPV within 12 months of the study and only include either of physical or sexual violence to measure the outcome variable. In the case of South Africa, they only report lifetime physical IPV.

Regarding factors associated with IPV, factors like being currently widowed/divorced/separated youth women were more likely to experience IPV than currently married youth women. Similar results have been found in studies conducted in Ethiopia [24], Canada [46], and Arkansas [25]. The possible reason might be that this high prevalence of IPV is expected to be the reason for being divorced and no longer living together or separated [47]. In addition, separated and divorced women were more likely to be abused by their previous intimate partners than married women [48].

In this study, the odds of experiencing IPV among youth women whose partner drank alcohol were higher as compared to youth women who had a partner who did not drink alcohol. This finding is consistent with studies conducted in Wolaita Sodo University, Ethiopia [26], low and middle-income countries [49], and SSA [45, 50]. This might be due to alcohol reducing self-control and decreasing the negotiation power of individuals because alcohol use directly affects cognitive and physical function. It also increases extravagancy, decreases martial satisfaction, and may contribute to conflict that could further lead to IPV [51].

Youth women who had witnessed inter-parental violence during childhood were more likely to experience IPV in their lifetime as compared to those youth women who had not witnessed inter-parental violence during childhood. This finding is supported by studies conducted in Wolaita Sodo University, Ethiopia [26], Nigeria [28], Nepal [29], and South America [52]. This might be explained by youth women exposed to observing violence during their early life developing attitudinal acceptance of violence and taking it as a normal way of relationships in their later life. Besides, this could be the intergenerational transmission of violence, which means that youths who witnessed inter-parental violence during childhood are more likely to exhibit violence in their own intimate relationships as a learned behavior from mothers to daughters [53–55].

The current study revealed that afraid of partner is associated with IPV among youth women. Youth women who were afraid of their partner had higher odds of experiencing IPV than those youth women who were not afraid of their partner. This is similar with studies conducted in Uganda [30, 31], and Nepal [29]. The possible explanation could be youth women afraid of their husband/partner reflects the imbalance of power between youth women and their husband/partner and it is commonly associated with experiencing a wide range of violent activities. Moreover, Youth women who were afraid of their partners might be less likely to say no to the sexual violence of their partners, which in turn aggravates the cycle of physical violence [56].

Furthermore, youth women residing in communities with a low proportion of educated youth women were less likely to experience IPV as compared to their counterparts. This finding is similar with a study done in Ethiopia [42]. The justification for this might be that youth women’s education by itself could not be sufficient to reduce the risk of IPV, rather counteracting traditional gender roles of male superiority and control over of their wives in culturally conservative areas is better [57]. This explanation is supported by a study conducted in South Africa, which states that the compensation hypothesis contends that the husband will use force to compensate for his inability to live up to the male-provider norm [58].

The main strength of this study was the use of weighted nationally representative data with a large sample, which makes it representative at national level. Moreover, this study has confirmed the contribution of factors at the individual and community level that influence IPV among youth women in Ethiopia. This is very important for specific intervention to achieve SDG goal 5. However, our study was not without limitations. Since IPV is a sensitive issue and social stigma is attached to it, social desirability biases might be expected. Furthermore, it was based on cross-sectional data; we are unable to establish a causal relationship between IPV and the identified independent variables.

## Conclusion

The magnitude of intimate partner violence among youth women in Ethiopia is relatively high as compared to the global estimate of IPV. Individual level variables such as currently widowed/divorced/separated women, having a partner who drank alcohol, witnessing inter-parental violence during childhood, and being afraid of partner have a positive significant association with experiencing intimate partner violence, whereas a community level variable such as low proportion of community level youth women's education is negatively associated with the outcome variable. Therefore, we recommend public health interventions like strengthening legislation on the purchase and sale of alcohol, providing legal protection for separated/divorced women, establishing effective legal response services for IPV, promoting gender equality, and providing psychological support for those who witnessed inter-parental violence during childhood to reduce IPV.

## Data Availability

All relevant data are within the manuscript and data is available publically access from the open databases. It can be accessed from www.measuredhs.com.

## Abbreviations

AOR: Adjusted odds ratio
CI: Confidence interval
EDHS: Ethiopia Demographic and Health Survey
IPV: Intimate partner violence
SSA: Sub-Saharan Africa
SDG: Sustainable Development Goal
WHO: World Health Organization

## References

[1] WHO. Sexual and reproductive health. 2021. https://www.who.int/reproductivehealth/topics/violence/sexual_violence/en/.

[2] WHO. Understanding and addressing violence against women: 2012. https://apps.who.int/iris/bitstream/handle/10665/77432/WHO_RHR_12.36_eng.pdf.

[3] Cadilhac DA, Sheppard L, Cumming TB, Thayabaranathan T, Pearce DC, Carter R (2015). The health and economic benefits of reducing intimate partner violence: an Australian example. BMC Public Health.

[4] Bitew T (2014). Prevalence and risk factors of depression in Ethiopia: a review. Ethiop J Health Sci.

[5] Brar SK, Beattie TSH, Abas M, Vansia D, Phanga T, Maseko B (2020). The relationship between intimate partner violence and probable depression among adolescent girls and young women in Lilongwe. Malawi Glob Public Health.

[6] Campbell JC (2002). Health consequences of intimate partner violence. The Lancet.

[7] Nur N (2014). Association between domestic violence and miscarriage: a population-based cross-sectional study among women of childbearing ages, Sivas, Turkey. Women Health.

[8] Udmuangpia T, Yu M, Laughon K, Saywat T, Bloom TL (2020). Prevalence, risks, and health consequences of intimate partner violence during pregnancy among young women: a systematic review. Pac Rim Int J Nurs Res.

[9] Anand E, Unisa S, Singh J (2017). Intimate partner violence and unintended pregnancy among adolescent and young adult married women in south Asia. J Biosoc Sci.

[10] Devries KM, Mak JY, Bacchus LJ, Child JC, Falder G, Petzold M (2013). Intimate partner violence and incident depressive symptoms and suicide attempts: a systematic review of longitudinal studies. PLoS Med.

[11] Kouyoumdjian FG, Calzavara LM, Bondy SJ, O’campo P, Serwadda D, Nalugoda F (2013). Intimate partner violence is associated with incident HIV infection in women in Uganda. AIDS.

[12] Jewkes RK, Dunkle K, Nduna M, Shai N (2010). Intimate partner violence, relationship power inequity, and incidence of HIV infection in young women in South Africa: a cohort study. The Lancet.

[13] Kafka JM, Moracco KE, Young B-R, Taheri C, Graham LM, Macy RJ (2021). Fatalities related to intimate partner violence: towards a comprehensive perspective. Inj Prev.

[14] Bonomi AE, Anderson ML, Rivara FP, Thompson RS (2009). Health care utilization and costs associated with physical and nonphysical-only intimate partner violence. Health Serv Res.

[15] Stöckl H, March L, Pallitto C, Garcia-Moreno C (2014). Intimate partner violence among adolescents and young women: prevalence and associated factors in nine countries: a cross-sectional study. BMC Public Health.

[16] Widom CS, Czaja S, Dutton MA (2014). Child abuse and neglect and intimate partner violence victimization and perpetration: a prospective investigation. Child Abuse Negl.

[17] Sardinha L, Maheu-Giroux M, Stöckl H, Meyer SR, García-Moreno C (2022). Global, regional, and national prevalence estimates of physical or sexual, or both, intimate partner violence against women in 2018. The Lancet.

[18] Ler P, Sivakami M, Monárrez-Espino J (2020). Prevalence and factors associated with intimate partner violence among young women aged 15 to 24 years in India: a social–ecological approach. J Interpers Violence.

[19] Maguele MS, Tlou B, Taylor M, Khuzwayo N (2020). Risk factors associated with high prevalence of intimate partner violence amongst school-going young women (aged 15–24years) in Maputo, Mozambique. PLoS ONE.

[20] Mthembu J, Mabaso M, Reis S, Zuma K, Zungu N (2021). Prevalence and factors associated with intimate partner violence among the adolescent girls and young women in South Africa: findings the 2017 population based cross-sectional survey. BMC Public Health.

[21] Maguele MS, Taylor M, Khuzwayo N (2020). Evidence of sociocultural factors influencing intimate partner violence among young women in sub-Saharan Africa: a scoping review. BMJ Open.

[22] Jesmin SS (2015). Married women’s justification of intimate partner violence in Bangladesh: examining community norm and individual-level risk factors. Violence Vict.

[23] Fahmy HH, Abd El-Rahman SI (2008). Determinants and health consequences of domestic violence among women in reproductive age at Zagazig District, Egypt. J Egypt Public Health Assoc.

[24] Chernet AG, Cherie KT (2020). Prevalence of intimate partner violence against women and associated factors in Ethiopia. BMC Womens Health.

[25] Shobe M, Christy K, Givens A, Hamilton L, Jordan S, Murphy-Erby Y (2011). Cohabitation and marital status: their relationship with economic resources and intimate partner violence. Int J Humanit.

[26] Adinew YM, Hagos MA (2017). Sexual violence against female university students in Ethiopia. BMC Int Health Hum Rights.

[27] Yaya S, Ghose B (2019). Alcohol drinking by husbands/partners is associated with higher intimate partner violence against women in Angola. Safety.

[28] Onanubi KA, Olumide AO, Owoaje ET (2017). Prevalence and predictors of intimate partner violence among female youth in an urban low-income neighborhood in Ibadan, South-West Nigeria. SAGE Open.

[29] Gautam S, Jeong H-S (2019). Intimate partner violence in relation to husband characteristics and women empowerment: evidence from Nepal. Int J Environ Res Public Health.

[30] Wandera SO, Kwagala B, Ndugga P, Kabagenyi A (2015). Partners’ controlling behaviors and intimate partner sexual violence among married women in Uganda. BMC Public Health.

[31] Kwagala B, Wandera SO, Ndugga P, Kabagenyi A (2013). Empowerment, partner’s behaviours and intimate partner physical violence among married women in Uganda. BMC Public Health.

[32] World Health Organization. Promoting gender equality to prevent violence against women: World Health Organization; 2009. https://apps.who.int/iris/bitstream/handle/10665/44098/9789241597883_eng.pdf.

[33] Ellsberg M, Arango DJ, Morton M, Gennari F, Kiplesund S, Contreras M (2015). Prevention of violence against women and girls: What does the evidence say?. The Lancet.

[34] Niolon PH. Centers for Disease C, Prevention. Preventing intimate partner violence across the lifespan: a technical package of programs, policies, and practices. Government Printing Office; 2017 6, 66.

[35] Kjaerulf F, Lee B, Cohen L, Donnelly P, Turner S, Davis R, et al. The 2030 agenda for sustainable development: a golden opportunity for global violence prevention. Springer; 2016. 10.1007/s00038-016-0887-8 27771749

[36] Croft TN, Marshall AMJ, Allen CK, et al. Guide to DHS statistics. Rockville: ICF; 2018.

[37] Azene ZN, Yeshita HY, Mekonnen FA (2019). Intimate partner violence and associated factors among pregnant women attending antenatal care service in Debre Markos town health facilities, Northwest Ethiopia. PLoS ONE.

[38] Trott CD, Harman JJ, Kaufman MR (2017). Women’s attitudes toward intimate partner violence in Ethiopia: the role of social norms in the interview context. Violence Against Women.

[39] Adhena G, Oljira L, Dessie Y, Hidru HD (2020). Magnitude of intimate partner violence and associated factors among pregnant women in Ethiopia. Adv Public Health.

[40] Tirore LL, Mulugeta A, Belachew AB, Gebrehaweria M, Sahilemichael A, Erkalo D (2021). Factors associated with anaemia among women of reproductive age in Ethiopia: multilevel ordinal logistic regression analysis. Matern Child Nutr.

[41] Ethiopia WB. The World Bank is helping to fight poverty and improve living standards in Ethiopia. Goals include promoting rapid economic growth and improving service delivery. 2019/2020. https://www.worldbank.org/en/country/ethiopia.

[42] Tiruye TY, Harris ML, Chojenta C, Holliday E, Loxton D (2020). Determinants of intimate partner violence against women in Ethiopia: a multi-level analysis. PLoS ONE.

[43] Kim JH (2019). Multicollinearity and misleading statistical results. Korean J Anesthesiol.

[44] Merlo J, Chaix B, Yang M, Lynch J, Råstam L (2005). A brief conceptual tutorial of multilevel analysis in social epidemiology: linking the statistical concept of clustering to the idea of contextual phenomenon. J Epidemiol Community Health.

[45] Wado YD, Mutua MK, Mohiddin A, Ijadunola MY, Faye C, Coll CVN (2021). Intimate partner violence against adolescents and young women in sub-Saharan Africa: Who is most vulnerable?. Reprod Health.

[46] Brownridge DA, Chan KL, Hiebert-Murphy D, Ristock J, Tiwari A, Leung W-C (2008). The elevated risk for non-lethal post-separation violence in Canada: a comparison of separated, divorced, and married women. J Interpers Violence.

[47] Seidu A-A, Aboagye RG, Ahinkorah BO, Adu C, Yaya S (2021). Intimate partner violence as a predictor of marital disruption in sub-Saharan Africa: a multilevel analysis of demographic and health surveys. SSM-Popul Health.

[48] Brownridge DA, Hiebert-Murphy D, Ristock J, Chan KL, Tiwari A, Tyler KA (2008). Violence against separated, divorced, and married women in Canada, 2004. J Divorce Remarriage.

[49] Greene MC, Heise L, Musci RJ, Wirtz AL, Johnson R, Leoutsakos J-M (2021). Improving estimation of the association between alcohol use and intimate partner violence in low-income and middle-income countries. Inj Prev.

[50] Greene MC, Kane JC, Tol WA (2017). Alcohol use and intimate partner violence among women and their partners in sub-Saharan Africa. Global Mental Health.

[51] Homish GG, Leonard KE (2007). The drinking partnership and marital satisfaction: the longitudinal influence of discrepant drinking. J Consult Clin Psychol.

[52] Lehrer JA, Lehrer VL, Lehrer EL, Oyarzun P. Sexual violence in college students in Chile. IZA Discussion Papers; 2007.

[53] Black DS, Sussman S, Unger JB (2010). A further look at the intergenerational transmission of violence: witnessing interparental violence in emerging adulthood. J Interpers Violence.

[54] Roberts AL, Gilman SE, Fitzmaurice G, Decker MR, Koenen KC (2010). Witness of intimate partner violence in childhood and perpetration of intimate partner violence in adulthood. Epidemiology.

[55] Kanwal Aslam S, Zaheer S, Shafique K (2015). Is spousal violence being “vertically transmitted” through victims? Findings from the Pakistan demographic and health survey 2012–13. PLoS ONE.

[56] Gill A (2004). Voicing the silent fear: South Asian women's experiences of domestic violence. Howard J Crim Just.

[57] Benebo FO, Schumann B, Vaezghasemi M (2018). Intimate partner violence against women in Nigeria: a multilevel study investigating the effect of women’s status and community norms. BMC Womens Health.

[58] Choi SYP, Ting K-F (2008). Wife beating in South Africa: an imbalance theory of resources and power. J Interpers Violence.

